# Combatting a silent killer – the importance of self-screening of blood pressure from an early age

**DOI:** 10.17179/excli2021-4140

**Published:** 2021-08-16

**Authors:** Sarosh Fatima, Samar Mahmood

**Affiliations:** 1 Department of Internal Medicine, Dr. Ruth K.M. Pfau Civil Hospital Karachi & Dow University of Health Sciences, Karachi, Pakistan

## ⁯⁯⁯⁯⁯

***Dear Editor,***

In the modern era, high blood pressure affects one in eight young adults aged between 20 and 40 years worldwide (Hinton et al., 2020[5]). The prevalence of hypertension (HTN) in young adults in the United States has been reported at 7.3 %, while developing countries like India show a slightly higher percentage, at 11-12 % (Hinton et al., 2020[5]; Dalal et al., 2016[2]). Owing to the 'sedentary shift' in lifestyles across decades, these statistics are only expected to rise. For instance, a South African survey of 2016 highlighted that the prevalence of HTN amongst people aged 25 to 34 years escalated dramatically from 15 % to 33 % and from 10.6 % to 27 % in males and females respectively, since 1998 (Jones et al., 2020[6]). Adiposity, hyperuricemia, hypercholesterolemia, and hypertriglyceridemia are the significant predictors of HTN in young people. Risk factors contributing to its development in this population include: physical inactivity, diabetes, obesity, consumption of processed foods that are high in fats, salt, or sugar, smoking, and drinking alcohol (Hinton et al., 2020[5]). In addition, the prevalence of secondary HTN is approximately 30 % in young hypertensive adults, stemming from various etiologies like renal or endocrine pathologies (Dalal et al., 2016[2]).

Hypertension is associated with a high mortality rate of 13.5 % annual deaths (Arima et al., 2011[1]) and mostly results in deaths from cardiovascular events like coronary heart disease, heart failure, and stroke (Hinton et al., 2020[5]). What is most worrying concerning young hypertensive adults is that: the earlier the age of onset of HTN, the greater the risk of development of cardiovascular disease (CVD) (Wang et al., 2020[7]). Recent research following individuals for six and a half years after onset of HTN concluded that people with earlier onset (under 45 years of age) had more than double the risk for CVD and mortality (Wang et al., 2020[7]). The study further proposed that the age of onset of HTN can assist in the stratification of CVD risk. In light of that, it is imperative to be aware of the actual age of hypertension at onset, rather than upon eventual diagnosis.

Hypertension is regarded as a silent killer because it may show no symptoms initially while silently causing sub-clinical organ damage in the body. Hence, it is even more essential to make a timely diagnosis and intervention in young individuals who are primarily uninformed about their hypertensive condition and often get diagnosed and treated much later than the older population (Hinton et al., 2020[5]; Wang et al., 2020[7]). This delay may be attributed to the fact that young people do not make frequent and regular visits to the primary care clinics and are generally found more negligent about their health in view of their preoccupations (Gao et al., 2013[4]). 

Self-screening of blood pressure could be an effective and practical intervention to this public health concern. It should be widely practiced, as it would aid the timely identification of hypertension. Although there is a paucity of data on the percentage of young people who undergo self-screening, amongst those involved in the practice, a median of 44 % of referred individuals found themselves newly diagnosed with hypertension (Fleming et al., 2015[3]). In developing countries with non-affording masses, government drives distributing blood pressure apparatus with guidance about operation would considerably further this cause. College and university-going students could be taught how to measure blood pressure and be followed up with regarding measurements of all their family members and directed to medical attention, as and where needed. Moreover, electronic blood pressure apparatus can be installed in the community sites like markets, malls, parks, worksites, colleges to make screening more accessible. Lastly, awareness programs organized at academic and work sites and on electronic and social media would catalyze the education of young people about the increasing incidence of HTN in their age group, its adverse outcomes, adoption of healthier lifestyles, and importance of regular self-screening of blood pressure. 

## Acknowledgements

None.

## Funding

None.

## Conflict of interest

None.
